# Oxidation state of Cu in silicate melts at upper mantle conditions

**DOI:** 10.1038/s41598-024-56538-9

**Published:** 2024-03-09

**Authors:** Xingcheng Liu, Lei Zhang, Sanyuan Zhu, Li Li, Xiaolin Xiong

**Affiliations:** 1 State Key Laboratory of Isotope Geochemistry, Guangzhou Institute of Geochemistry, Chinese Academy of Sciences, Guangzhou, 510640 China; 2 University of Chinese Academy of Sciences, Beijing, 100049 China; 3 State Key Laboratory of Organic Geochemistry, Guangzhou Institute of Geochemistry, Chinese Academy of Sciences, Guangzhou, China

**Keywords:** Geochemistry, Economic geology

## Abstract

Beyond its economic value, copper (Cu) serves as a valuable tracer of deep magmatic processes due to its close relationship with magmatic sulfide evolution and sensitivity to oxygen fugacity (fO_2_). However, determining Cu’s oxidation state (+ 1 or + 2) in silicate melts, crucial for interpreting its behavior and reconstructing fO_2_ in the Earth’s interior, has long been a challenge. This study utilizes X-ray Absorption Near Edge Structure spectroscopy to investigate the Cu oxidation state in hydrous mafic silicate melts equilibrated under diverse fO_2_ (− 1.8 to 3.1 log units relative to the Fayalite–Magnetite–Quartz buffer), temperature (1150–1300 °C), and pressure (1.0–2.5 GPa) conditions. Our results reveal that Cu predominantly exists as Cu^+^ across all fO_2_ conditions, with a minor Cu^2+^ component. This dominance of Cu^+^ persists even in relatively oxidized melts, highlighting its limited sensitivity to fO_2_ under upper mantle conditions. This significantly constrains the utility of Cu as an oxybarometer in hydrous silicate melts in the deep Earth. However, our findings suggest that Cu isotopes primarily reflect the interplay of sulfide segregation/accumulation during magmatic differentiation, shedding light on these fundamental processes in Earth’s interior.

## Introduction

Copper, beyond its critical role as a mineral resource, serves as a sensitive tracer for unraveling deep magmatic processes^1–8^. Its geochemical behavior is inextricably linked to the fate of magmatic sulfide, as Cu exhibits pronounced incompatibility within silicate minerals^7,9,10^. This intimate relationship makes Cu a valuable proxy for tracking sulfide evolution, which is highly sensitive to changes in oxygen fugacity (fO_2_) within the magma reservoir. Furthermore, Cu isotopes can undergo significant fractionation during magmatic differentiation involving sulfides, further amplifying its potential as a geochemical recorder^1,4^. Consequently, the combined analysis of Cu content and isotope variations in igneous rocks offers invaluable insights into magma evolution and the fO_2_ conditions in the deep Earth.

The multivalent nature of Cu allows it to exist as Cu^+^ and Cu^2+^ in silicate melts and fluids^7,11–13^. Its valence state profoundly impacts its diffusion, partitioning behavior, and Cu isotope fractionation during magmatic evolution^1,4,10,14,15^. Cu^+^ exhibits significantly higher diffusivity than Cu^2+^, likely due to its smaller ionic radius and weaker interaction with the melt structure^15,16^. This order-of-magnitude difference in mobility compared to sulfur and chlorine suggests that the Cu^+^/Cu^2+^ ratio could influence Cu’s partitioning behavior, particularly during disequilibrium magmatic and hydrothermal processes. Previous studies indicate the average Cu valence state in silicate melts under upper mantle conditions ranges from + 1 to + 2, increasing with elevated fO_2_^7^. Wood and Kiseeva^17^ further suggest that the Cu^+^/Cu^2+^ ratio, alongside FeO content, significantly affects Cu partitioning between sulfide and silicate melts. Given the dominance of Cu^+^ in magmatic sulfides, the equilibrium Cu isotope fractionation factor between sulfide and silicate melts can be substantial, reaching values up to ~ 2‰ (e.g., Liu et al.^1^). Similarly, the oxidation state of Cu in aqueous fluids is predominantly Cu^+^ at high temperatures^11,18^, potentially explaining Cu isotope fractionation between fluids and silicate melts^19^. This dependence on fO_2_ raises the intriguing possibility of using the Cu^+^/Cu^2+^ ratio in silicate melts as a novel oxybarometer (a tool for inferring fO_2_).

Previously, determining the valence state of Cu in silicate melts relied on analyzing the slope of its solubility data plotted against fO_2_. This approach relied on the relationship: Cu^metal^ + n/2 O_2_ = CuO_n_^melt^, where logCu solubility (ppm) = n/2 log fO_2_ + C. A slope of 0.5 indicated Cu^2+^ dominance (n = 1), while 0.25 suggested Cu^+^ (n = 0.5). However, this method faced limitations. Melt composition and temperature significantly influenced the slope, introducing ambiguities and inaccuracies in valence state determination. XANES (X-ray Absorption Near Edge Structure) emerges as a powerful tool that overcomes these limitations. By analyzing the Cu K-edge spectrum, it provides direct and quantitative measurements of Cu’s oxidation state, surpassing the indirect and often ambiguous inferences drawn from the slope method. This precision allows researchers to not only confirm the dominant valence state but also identify subtle variations and even the coexistence of multiple oxidation states within a single melt, shedding light on a previously hidden dimension of Cu behavior in silicate melts. The application of XANES for determining Cu oxidation state in silicate melts is a recent development, exemplified by the work of Miller et al.^20^. Their research demonstrated that Cu predominantly exists as Cu^+^ in silicate melts under terrestrial conditions, focusing on anhydrous melts (mostly Fe-free) at ambient pressure and pressures up to 2.5 GPa, as well as the examination of hydrous haplogranitic melts under 1.0 GPa.

Hydrous basalts, which form in the mantle wedge at subduction zones, are critical for studying magmatic processes and tracing fO_2_ using Cu. However, the oxidation state of Cu in these silicate melts, particularly in the presence of iron and under high pressures, remains poorly understood. To address this gap, we employ XANES spectroscopy to determine the Cu^+^/ΣCu ratio (where ΣCu = Cu^+^ + Cu^2+^) for silicate glasses quenched from both Fe-bearing and Fe-free hydrous basaltic melts. These melts were equilibrated under diverse fO_2_ conditions (− 1.8 to 3.1 log units relative to the fayalite–magnetite–quartz buffer, FMQ), at temperatures ranging from 1150 to 1300 °C, and under pressures from 1.0 to 2.5 GPa. By elucidating the oxidation state of Cu in these melts under upper mantle conditions, this study holds the potential to refine our understanding of magmatic processes and fO_2_ variations within the Earth’s interior.

## Results and discussion

### Run products

Seven piston cylinder experiments (detailed in Table 1) employed three synthetic silicate glasses (Table S1) as starting materials: four MORB-like, two Di_70_An_30_, and one komatiitic. Despite mineral crystallization occurring in five runs (20–33 wt%), segregation of silicate melt pools at the capsule top facilitated XANES analysis (Fig. S1). This favorable distribution, combined with low Cu partition coefficients between mafic melts and minerals (D < 0.2)^7^, minimized the influence of mineral-hosted Cu on XANES spectra.

**Table 1:** Experimental conditions and run products.

Run No	Starting material	T (°C)	P (GPa)	Run duration (h)	Initial H_2_O (wt%)	fO_2_ Buffer	Estimated fO_2_ (∆ FMQ)	Run products (wt%)	Cu in melt (ppm)
Cu-6	MORB	1150	1.0	72	5.1	Not bufferd	–	gl (76), cpx (21), spl (3)	4885 (76)
Cu-8	MORB	1150	1.0	72	6.1	MnMnO	3.1	gl (100)	6320 (320)
Cu-45	MORB	1150	1.0	74	5.2	Not bufferd	–	gl (100)	5848 (284)
MORB-L2	MORB	1250	2.5	37	5.2	NNO	− 0.6	gl (80), cpx (20)	420 (9)
Cu-52	Di_70_An_30_	1230	1.0	49	5.0	FMQ	–	gl (67), cpx (33)	4777 (228)
Cu-47	Di_70_An_30_	1240	1.0	48	5.0	Not bufferd	–	gl (80), cpx (20)	610 (21)
Cu-50	Komatiite	1300	1.0	27	1.0	Graphite	− 1.8	gl (73), ol (19), opx (7), spl (1)	281 (8)

Cu concentrations in melt, except for runs Cu-8 and Cu-45, have been reported in Liu et al.^7^. fO_2_ buffers used were FMQ (fayalite–magnetite–quartz), NNO (Ni-NiO), and MnMnO (MnO-Mn_3_O_4_). In run Cu-52, the FMQ buffer failed due to the absence of fayalite in the buffer assemblage after experiment. For runs Cu-8, MORB-L2, and Cu-50, fO_2_ was calculated based on melt water activity following methods in Xu et al.^23^ and Burnham^24^. An estimated relative fO_2_ sequence for the unbuffered runs, based on Cu solubility, is provided: Cu-8 > Cu-45 > Cu-6 > Cu-52 > Cu-47 > MORB-L2 > Cu-50 (detailed in the text). The modal abundances of these products were determined through mass balance calculations, as detailed in Liu et al.^7^

*Gl* glass, *Ol* olivine, *Opx* orthopyroxene, *Cpx* clinopyroxene, *Spl* spinel.

Compositions of the quenched glasses are presented in Table 2; refer to Liu et al.^7^ for detailed mineral compositions. These hydrous melts exhibit basaltic characteristics, although some minor compositional deviations from the starting materials may arise due to olivine and pyroxene crystallization. Estimated H_2_O contents, derived from the difference between EMP analytical totals (which exclude volatiles) and 100%, range from 5.7 to 11.5 wt%. These values often exceed those expected from the H_2_O contents added to the starting materials, likely due to H_2_ diffusion from the buffer materials and mineral crystallization^7^.

**Table 2:** Composition (wt%) and Cu concentrations (ppm) in quenched glasses.

Run No	Cu-6	Cu-8	Cu-45	MORB-L2	Cu-52	Cu-47	Cu-50
EMP (n)	30	11	15	10	10	10	13
SiO_2_	49.43 (0.29)	46.66 (0.22)	46.89 (0.23)	49.49 (0.16)	46.53 (0.24)	47.26 (0.31)	48.36 (0.64)
TiO_2_	1.03 (0.07)	1.06 (0.09)	1.21 (0.08)	1.84 (0.05)	0.03 (0.01)	0.03 (0.03)	0.28 (0.05)
Al_2_O_3_	15.59 (0.11)	14.12 (0.11)	13.64 (0.11)	16.62 (0.08)	15.00 (0.13)	13.51 (0.26)	14.68 (0.27)
FeO_T_	8.23 (0.17)	9.46 (0.19)	9.46 (0.17)	2.03 (0.03)	0.14 (0.03)	0.02 (0.02)	2.99 (0.18)
MnO	0.02 (0.01)	0.02 (0.01)	0.03 (0.02)	0.02 (0.02)	0.02 (0.02)	0.01 (0.01)	0.10 (0.02)
MgO	5.39 (0.09)	6.37 (0.14)	6.23 (0.09)	5.62 (0.05)	6.10 (0.13)	7.55 (0.16)	13.41 (0.45)
CaO	8.98 (0.13)	11.20 (0.07)	11.01 (0.09)	9.32 (0.09)	21.43 (0.17)	21.39 (0.17)	12.89 (0.26)
Na_2_O	3.18 (0.11)	2.71 (0.14)	2.59 (0.06)	2.88 (0.07)	0.07 (0.02)	0.76 (0.04)	1.10 (0.04)
K_2_O	0.26 (0.02)	0.22 (0.02)	0.23 (0.01)	0.29 (0.02)	0.05 (0.01)	0.12 (0.01)	0.10 (0.02)
P_2_O_5_	0.30 (0.02)	0.27 (0.03)	0.20 (0.03)	0.34 (0.03)	0.02 (0.01)	0.02 (0.01)	0.12 (0.02)
NiO	0.02 (0.01)	0.02 (0.01)	0.05 (0.03)	0.02 (0.02)	0.02 (0.02)	0.01 (0.00)	0.02 (0.02)
Cr_2_O_3_	0.03 (0.01)	0.03 (0.02)	0.02 (0.01)	0.01 (0.02)	0.03 (0.02)	0.02 (0.02)	0.19 (0.04)
CuO	0.51 (0.04)	0.79 (0.04)	0.43 (0.03)	0.05 (0.02)	0.47 (0.04)	0.07 (0.04)	0.04 (0.03)
Total	92.92 (0.50)	92.91 (0.46)	91.94 (0.43)	88.52 (0.34)	89.88 (0.46)	90.74 (0.42)	94.28 (0.62)
H_2_O	7.1	7.1	8.1	11.5	10.1	9.3	5.7
Optical basicity	0.564	0.575	0.570	0.541	0.562	0.568	0.574
LA-ICP-MS (n)	4	n.a	8	3	7	6	7
Cu (ppm)	4885 (76)	n.a	5848 (284)	420 (9)	4777 (228)	610 (21)	281 (8)

Glass compositions, except for runs Cu-8 and Cu-45, are reported in Liu et al.^7^. H_2_O contents were estimated by difference, calculated as 100% minus the sum of all other oxide components. Optical basicity values were calculated as described in Miller et al.^20^. n: number of analyses. Numbers in brackets represent ± 1σ standard deviation. n.a. = not analyzed.

### Cu contents in melts and fO_2_ estimation

Utilizing Pt_95_Cu_05_ alloy capsules, the measured Cu contents in the silicate melts represent their apparent solubilities. Previous studies have established that the solubility of Cu in mafic melts rises with temperature and fO_2_, with minimal pressure dependence at upper mantle conditions^7,10,12,13,21,22^. Notably, Cu solubility exhibits minimal variation among basaltic, andesitic, and dacitic melts at comparable temperatures and fO_2_^21^. In this study, Cu contents in silicate melts were measured using LA-ICP-MS and EPMA, ranging from 281 to 6320 ppm (Tables 1 and 2).

Accurate fO_2_ values were determinable for runs Cu-8, MORB-L2, and Cu-50, where fO_2_ buffer control was successfully established. Methods outlined by Xu et al.^23^ and Burnham^24^ estimated fO_2_ in these runs, yielding a range from ΔFMQ − 1.8 to ΔFMQ + 3.1. Subsequently, the fO_2_ conditions in unbuffered runs were estimated based on their respective melt Cu contents, as observed variations in Cu content primarily arise from differences in temperature and fO_2_. The higher Cu contents observed in the 1150 °C runs (Cu-6, 8, 45) compared to those in the 1230–1300 °C runs provide evidence of their elevated fO_2_ conditions, supporting the fO_2_ estimations obtained for the successfully buffered runs (Table 1). This trend is further validated by comparing the Cu contents across these runs: 6320 ± 320 ppm (ΔFMQ + 3.1) in Cu-8, 420 ± 9 ppm (ΔFMQ − 0.6) in MORB-L2, and 281 ± 8 ppm (ΔFMQ − 1.8) in Cu-50. Therefore, the melt Cu content can serve as a proxy indicator of fO_2_ conditions in the unbuffered runs.

In the subset of 1150 °C runs utilizing MORB as starting material, Cu-6 (4885 ± 76 ppm Cu) and Cu-45 (5848 ± 284 ppm Cu) likely have slightly lower fO_2_ than Cu-8 (6320 ± 320 ppm Cu at ΔFMQ + 3.1), as evidenced by their slightly lower, yet comparable, Cu contents. Similarly, Cu-52 (4777 ± 228 ppm Cu) at 1230 °C is expected to have a lower fO_2_ than Cu-6 (1150 °C) but significantly higher than MORB-L2 (ΔFMQ − 0.6) due to their comparable Cu contents. Additionally, Cu-47 exhibits a slightly higher fO_2_ than MORB-L2. Consequently, the estimated relative fO_2_ sequence for the unbuffered runs is as follows: Cu-8 (ΔFMQ + 3.1) > Cu-45 > Cu-6 > Cu-52 > Cu-47 > MORB-L2 (ΔFMQ − 0.6) > Cu-50 (ΔFMQ − 1.8).

### Capsule influence minimized on Cu K-edge XANES

This study utilized Pt_95_Cu_05_ alloy capsules in piston cylinder experiments to serve as Cu source and prevent Cu loss^7^. However, their significantly higher Cu content (~ 5 wt%) compared to the silicate melts (200–6000 ppm) posed a potential challenge: misleading Cu K-edge XANES spectra due to unwanted contributions from the capsule walls. To address this concern, we implemented a focused beam strategy using a slit collimation system. This strategy confined the synchrotron X-ray beam to a diameter less than 1 mm during non-focusing mode, selectively probing the central region of the glass sample and minimizing the influence of Cu from the surrounding capsule wall.

**Figure 1:**
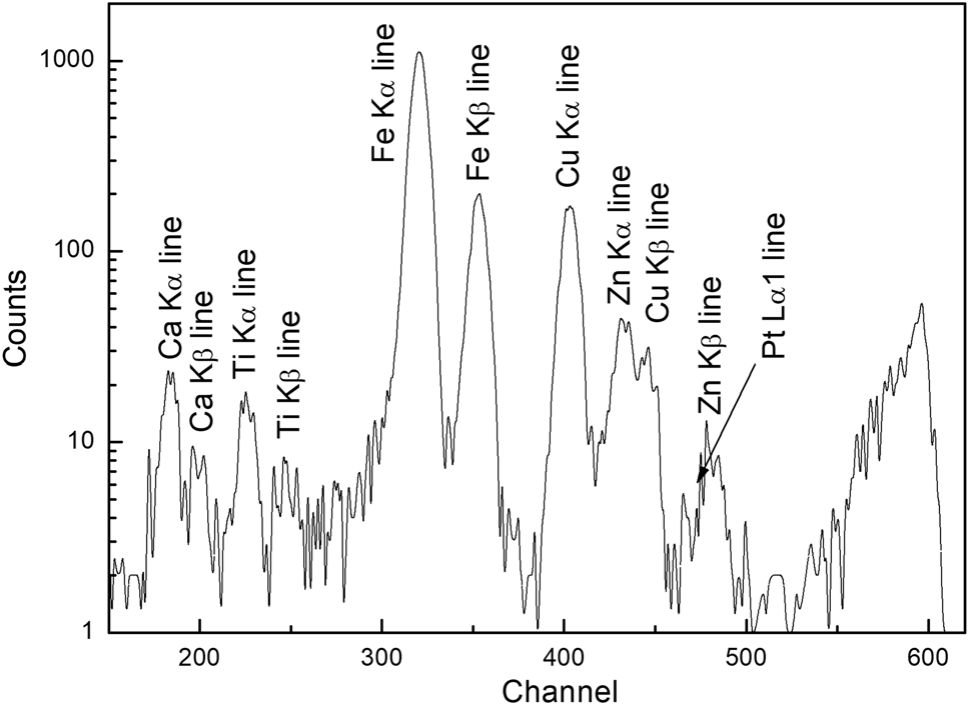
Fluorescence spectrum of sample Cu-6, indicating limited influence from the Pt_95_Cu_05_ alloy capsule during the XANES measurement.

**Figure 2:**
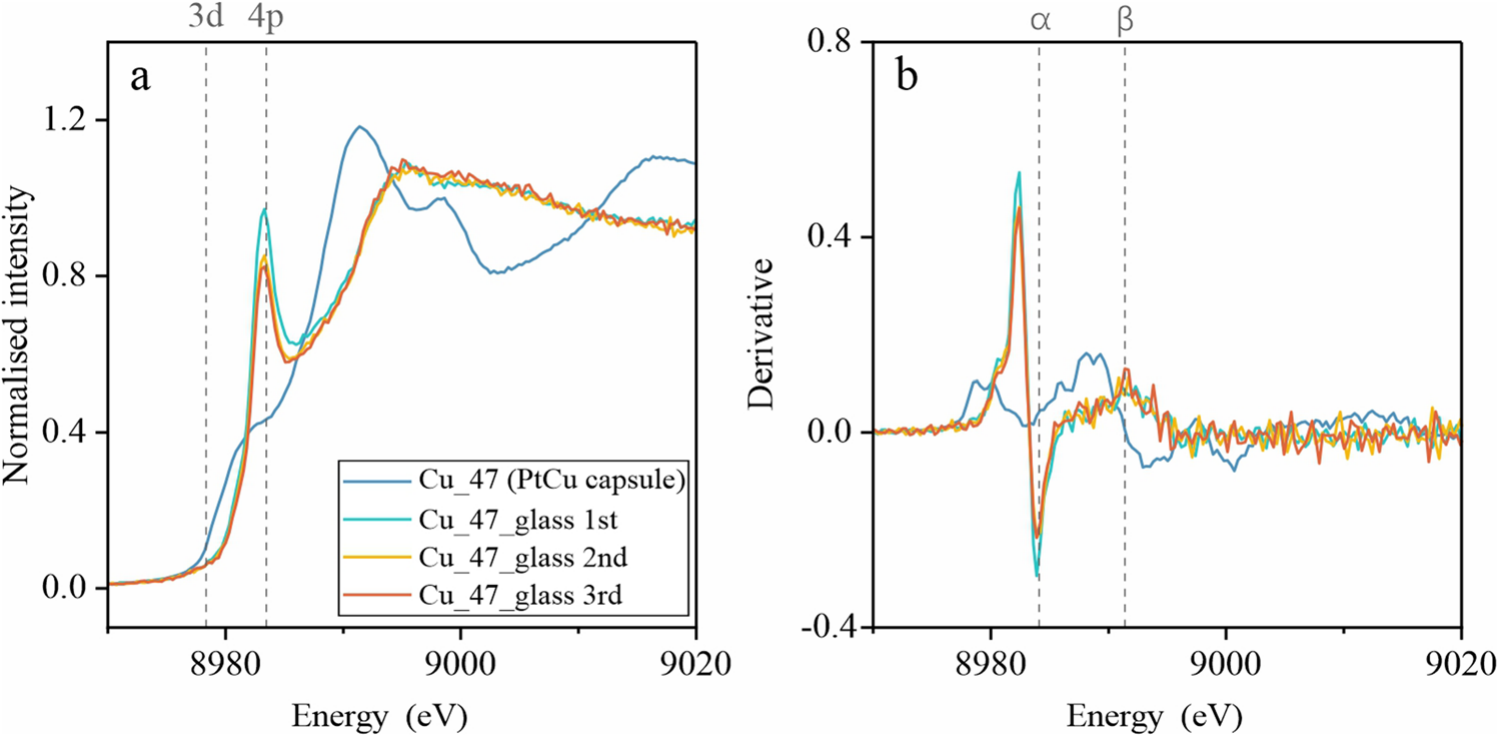
(**a**) Cu K-edge XANES spectra and (**b**) corresponding derivative spectra for the Pt_95_Cu_05_ alloy capsule and the enclosed quenched silicate glass from run Cu-47. This comparison allows for examination of the influence of the capsule material on the Cu signal in the glass and ensures the integrity of the data acquired from the sample. The XANES spectra were acquired three times on the same glass location to evaluate potential beam damage during the analysis.

The effectiveness of this approach is evident in both the minimal Pt fluorescence intensity and the dominant Cu fluorescence intensity observed in the X-ray spectrum (Fig. 1). While the Pt:Cu ratio in the capsule is 19:1, the Cu fluorescence intensity (200–300 counts) far exceeds the expected contribution from the capsule material. This confirms that the focused beam successfully targeted the glass sample, minimizing contributions from the Cu-rich capsule wall. Furthermore, we obtained the XANES spectra of both the capsule wall and quenched silicate glasses in Cu-47 for comparison (Fig. 2). The results reveal distinct spectra for the capsule and glasses, indicating that the influence of the capsule wall on XANES can be readily discerned. Consequently, the prominent Cu peak clearly originates from the glass sample itself, providing reliable data for investigating its Cu valence states and coordination.

### Assessment of beam damage

Beam damage in XANES can alter oxidation states through thermal effects and photo-induced oxidation/reduction. Previous studies observed oxidation of Cu in most samples and reduction of highly oxidized ones (ΔFMQ > 7.4) due to this phenomenon^20^. To evaluate potential beam damage in this study, we employed time-dependent monitoring and spectral comparisons at the XANES beamline. We acquired XANES spectra at the same glass location in run Cu-47 three times to monitor for potential changes during analysis. The spectra retained the same overall features throughout, although minor fluctuations, particularly in the pre-edge peak intensity, were observed (Fig. 2). Comparing spectra collected at different times and locations on the same sample further supported these observations, suggesting minimal impact of beam damage on Cu oxidation state in the silicate melts. Notably, we observed no significant oxidation or reduction attributable to beam damage in our samples. This minimal beam damage effect confirms the reliability of our XANES results in accurately reflecting the Cu oxidation state within the silicate melts. This supports the conclusions drawn from our analysis regarding Cu behavior at upper mantle conditions.

### Oxidation state of Cu in silicate melts

**Figure 3:**
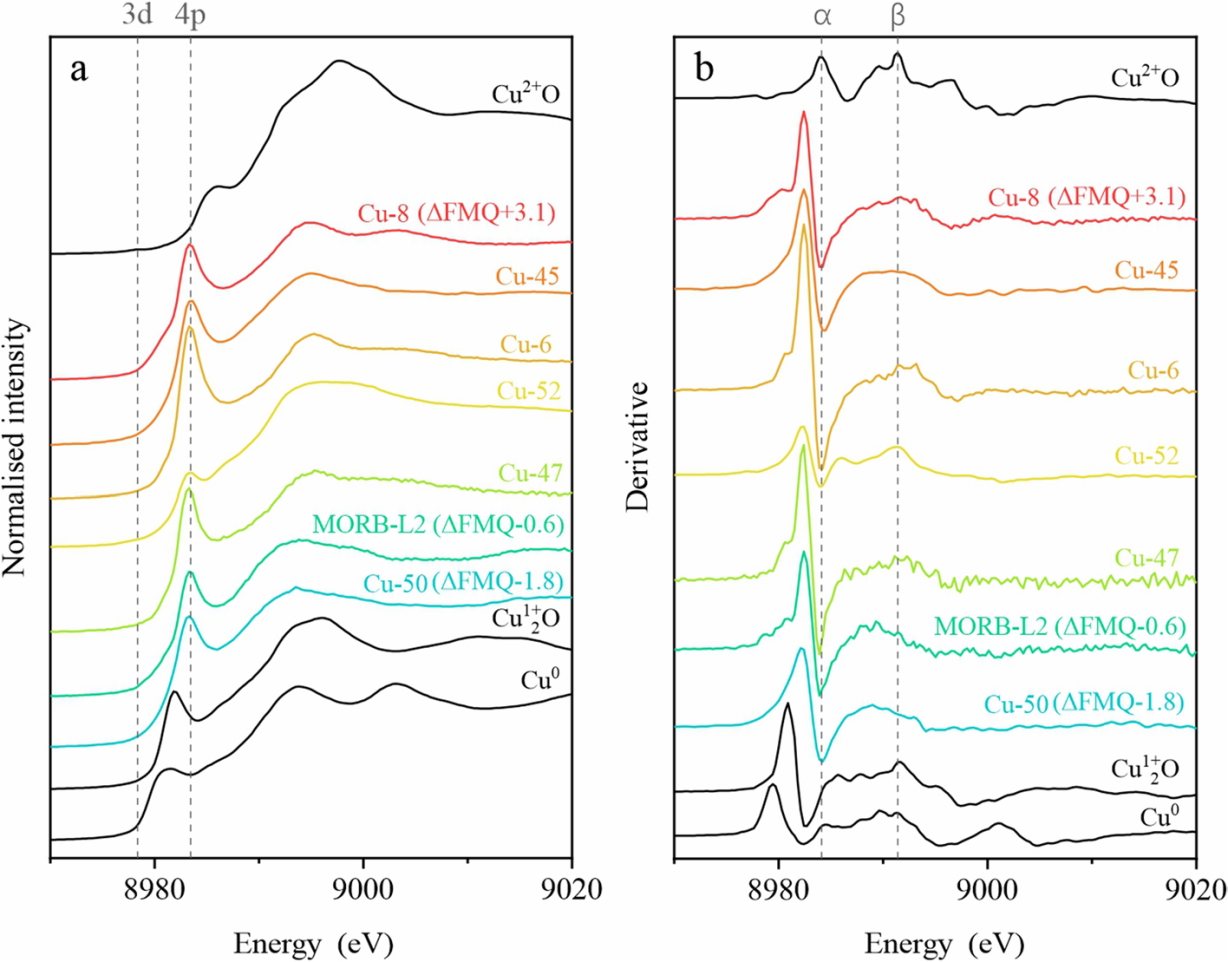
Comparison of Cu K-edge XANES and derivative spectra for Cu reference standards and quenched hydrous basaltic glasses. The pre-edge peak in the derivative spectra highlights Cu^+^ as the dominant valence state in the glasses. Spectra are organized by decreasing fO_2_ (top to bottom).

Figure 3 exhibits the normalized Cu K-edge XANES spectra of reference standards and quenched glass samples, revealing the dominance of Cu^+^ in these hydrous basaltic melts. Key to this determination is the first peak of the derivative spectrum: 8979.4 eV for Cu^0^, 8980.9 eV for Cu^+^_2_O, and 8984.0 eV for Cu^2+^O (Fig. 3b). Notably, Fig. 3a shows a characteristic pre-edge peak centered around 8983.4 eV in all melt glasses, confirming Cu^+^ presence due to the 1s → 4p electron excitation^11,20,25^. This peak weakens with increasing coordination number, reflecting orbital interactions with surrounding ligands^20^. While Cu^+^ in silicate melts and Cu^+^ in the Cu_2_O standard share the same oxidation state, their specific XANES features differ due to distinct chemical environments and coordination geometries. Conversely, Cu^2+^ exhibits a weak peak around 8979 eV due to the dipole-forbidden 1s → 3d transition, absent in Cu^+^ due to filled 3d orbitals^20,25^. Remarkably, even across a wide range of fO_2_ conditions (ΔFMQ − 1.8 to ΔFMQ + 3.1), no systematic correlations between Cu oxidation state and fO_2_ were observed in our study. Figure 3 clearly illustrates this lack of dependence, and the unexpectedly weak intensity in run Cu-52 (with conditions similar to the higher-intensity run Cu-47 but under a much more reduced condition) further reinforces this finding. This finding deviates from Miller et al.^20^, who observed increasing pre-edge intensity with decreasing fO_2_.

While Miller et al.^20^ observed features indicating Cu^2+^ in oxidized samples (ΔFMQ > 5.1), our spectra lack the prominent 1s → 3d transition peak at 8979 eV and the shoulder attributed to Cu^2+^O. However, faint echoes of these features appear in the most oxidized runs (Cu-8, 45, 6, and 52) as weak flat peaks near ~ 8991 eV in the derivative spectra, potentially corresponding to the α and β peaks observed by Miller et al.^20^ at ~ 8984.1 eV and ~ 8991.4 eV, respectively. These peaks, attributed to Cu^2+^O in an octahedral coordination environment, are significantly less pronounced in our samples compared to the CuO standard, suggesting a very minor contribution of Cu^2+^O alongside the overwhelming dominance of Cu^+^.

The peak at 8978.8 eV, associated with Cu^0^, only appears in the derivative spectra of the alloy capsule in run Cu-47 (Fig. 2b), along with the standard (Fig. 3). These results collectively indicate Cu^+^ as the dominant valence state in the silicate melts, accompanied by a minor Cu^2+^ component. This aligns with the estimation using the empirical equation from Miller et al.^20^, yielding Cu^+^/ΣCu values of 0.903 in Cu-8 (ΔFMQ + 3.1), 0.983 in MORB-L2 (ΔFMQ − 0.6) and 0.993 in Cu-50 (ΔFMQ − 1.8).

**Figure 4:**
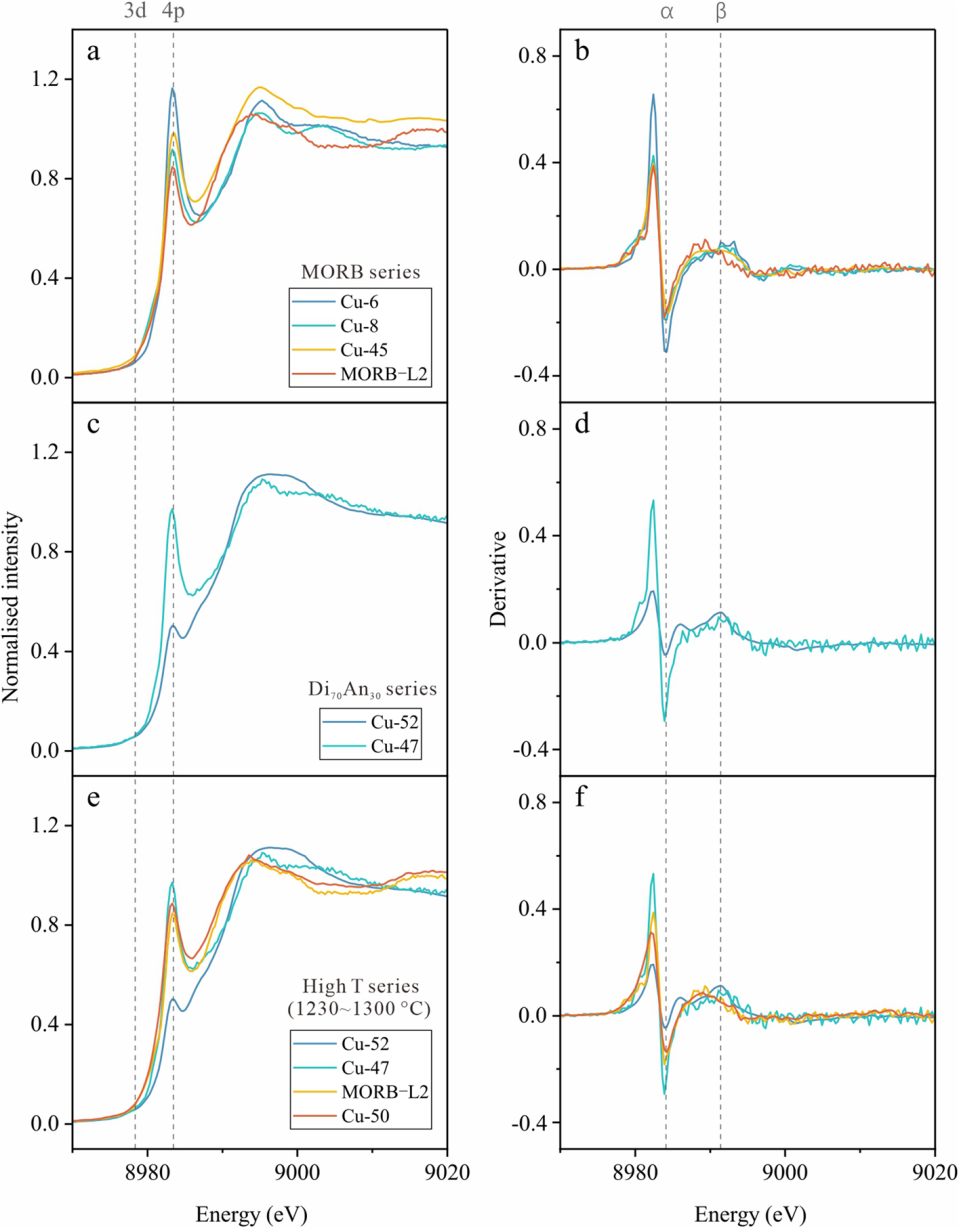
Comparison of Cu K-edge XANES and derivative spectra for glasses with different starting materials (MORB, Di_70_An_30_, komatiite) under varying pressure (1.0–2.5 GPa), temperature (1150–1300 °C), and fO_2_ conditions (ΔFMQ − 1.8 to 3.1). MORB and Di_70_An_30_ series: fO_2_ range approximately ΔFMQ − 0.6 to 3.1.

As shown in Fig. 4, temperature, pressure, and melt composition appear to exert minimal influence on Cu’s oxidation state within our experimental range, echoing recent findings that Cu^+^/ΣCu weakly depends on composition, with a slight preference for Cu^2+^ in more mafic melts^20^. They also found that increasing temperature stabilizes Cu^+^, while increasing pressure had little effect on Cu^+^/ΣCu in mafic melts but preferentially stabilized Cu^2+^O in granite melts. Importantly, arc basalts that form at subduction zones are predominantly produced through fluid-induced melting of the mantle wedge. The quenched glasses in this study, which mimic natural hydrous basaltic magmas under sub-arc mantle conditions, provide a more direct relevance to the interpretation of natural samples. By emphasizing the dominance of Cu^+^ in hydrous arc basalts under these conditions, our work not only corroborates established findings but also contributes valuable insights into the behavior of Cu in such geological settings.

### Geological implications

Our XANES analysis, in agreement with recent studies^20^, reveals Cu^+^ as the dominant oxidation state in hydrous mafic melts under upper mantle conditions, exceeding 90% in most cases even under relatively oxidized conditions. This prevailing dominance of Cu^+^ across a broad spectrum of fO_2_ values significantly diminishes the sensitivity of the Cu^+^/Cu^2+^ ratio as an indicator for terrestrial fO_2_. As a result, the effectiveness of the Cu^+^/ΣCu ratio as an oxybarometer to monitor the genesis and evolution of hydrous basaltic magmas within subduction zones is compromised, as the Cu^+^/ΣCu ratio only fluctuates from approximately 90% to 98%. However, it unlocks new avenues for understanding magmatic processes through Cu isotopes. Previous studies have demonstrated that Cu isotope fractionation can occur during: (a) sulfide-silicate melt partitioning, where Cu preferentially concentrates in sulfide, leading to isotopically heavier equilibrium silicate melts^1,4^; (b) fluid-silicate melt partitioning, where exsolved chlorine-bearing fluids have higher δ^65^Cu than the residual magmas^19^; and (c) electron-exchange-driven fractionation, where changes in Cu oxidation state, such as Cu^+^ to Cu^2+^, can result in isotopic fractionation^26^. Crucially, our finding of Cu^+^’s dominance under diverse fO_2_ conditions suggests that Cu oxidation state changes are unlikely to be the primary driver of Cu isotope variations in natural samples. Instead, the observed isotopic signatures likely reflect the interplay of sulfide segregation/accumulation or fluid exsolution during magmatic differentiation. For example, given the affinity of Cu^+^ for sulfide phases, its geochemical behavior is closely linked to the evolution of sulfur during magma generation, ascent, and cooling^1^. Under upper mantle conditions, Cu is primarily hosted in Cu-poor monosulfide solid solutions or sulfide melts, with minimal partitioning into other phases^1,2,7^. This accounts for the limited isotopic fractionation observed during partial melting of the mantle, even in the presence of sulfide residues^1^. However, as magmas evolve and cool at lower crustal levels, the formation of Cu-rich sulfides leads to substantial isotopic fractionation, with isotopically heavier Cu preferentially incorporated into these sulfide phases. Additionally, chlorine-rich fluid saturation at depth can also lead to lighter δ^65^Cu in residual phases. By decoupling redox variations from isotopic signatures, we can gain clearer insights into the evolution of sulfur within magmatic systems, which is not solely related to redox changes^23^. This newfound understanding can illuminate critical aspects of magmatic differentiation, including the formation of ore deposits and the behavior of volatile elements.

## Methods

### Starting materials

This study examined the valence state of Cu in different silicate melts using three mafic compositions—komatiite, mid-ocean ridge basalt (MORB), and Fe-free Di_70_An_30_ (diopside-anorthite) (Table S1). Reagent-grade oxides (SiO_2_, Al_2_O_3_, Fe_2_O_3_, MgO, TiO_2_, MnO, NiO, P_2_O_5_ and Cr_2_O_3_) and carbonates (CaCO_3_, Na_2_CO_3_, and K_2_CO_3_) were mixed, ground under acetone, and successively sintered at 1000 °C for ten hours and fused at 1500 °C for two hours in platinum crucibles to remove CO_2_ and ensure chemical homogeneity. Subsequently, the crucibles were rapidly cooled by immersion in purified water, and the quenched glass was finely ground to yield homogeneous glass powder. Two rounds of this fusion-grinding process were employed to achieve complete decarbonation and enhance melt consistency. For more details, refer to Liu et al.^7^, where a portion of the Cu solubility results were previously reported.

### Sample capsules and fO_2_ control

We employed the same experimental setup as described in Liu et al.^7^, utilizing Pt_95_Cu_05_ alloy capsules in two sizes: a smaller sample container (ID: 2.7 mm, OD: 3.0 mm) and a larger capsule for sample and oxygen fugacity solid buffer (ID: 4.7 mm, OD: 5.0 mm). Each capsule, except for Cu-50 with 1 wt% H_2_O, received approximately 15 mg of initial silicate powder and 5–6 wt% H_2_O before welding. The added H_2_O facilitates equilibration and promotes the formation of crystal-free basaltic glasses during the experiments. Notably, in Cu-50, the sample capsule was loaded into a graphite capsule within a larger alloy capsule, with one end welded and the other crimped for communication with the graphite. Following welding, all capsules underwent drying to confirm no leaks. The alloy capsule also serves as the source of Cu. With a high mass ratio of the capsule (12–16 times the silicate charge), the alloy capsule acts as a buffer, maintaining a constant Cu activity or concentration in each phase of the silicate charge under specific P–T-fO_2_ conditions. This approach guarantees Cu homogeneity within the melts and circumvents the disequilibrium issues commonly associated with the “Cu-loss problem”.

Oxygen fugacity of primitive arc basalts spans a wide range, typically extending from ΔFMQ-2.0 to ΔFMQ + 3.5, with most clustering between FMQ and ΔFMQ + 2.0^3,27^. To replicate this diversity in the study, a conventional double-capsule technique utilizing Fayalite–Magnetite–Quartz (FMQ), Ni-NiO (NNO), and MnO-Mn_3_O_4_ (MnMnO) buffers was employed to control the experimental fO_2_, following the methodology outlined in Liu et al.^7^. In this configuration, a sealed sample capsule containing the silicate powder is welded into an outer alloy capsule. The space between the capsules is then filled with the specific buffer material and H_2_O. It’s important to note that the actual fO_2_ should be slightly lower than the imposed buffer due to the H_2_O activity in the silicate melt being less than unity^23^. On the other hand, three unbuffered experiments were conducted using a single sample capsule placed inside an MgO tube with crushable spacers and MgO powder filling the remaining space. In these runs, the fO_2_ is believed to be primarily imposed by the starting material and the cell assembly.

### Piston cylinder experiments

Primitive arc basalts form under a range of temperature and pressure conditions, typically spanning 1150–1350 °C and 0.8–2.1 GPa, respectively^27^. To simulate these conditions and investigate Cu behavior in arc basalts, our experiments employed high pressures (1.0–2.5 GPa) and temperatures (1150–1300 °C) with durations of 27–74 h using end-loaded piston-cylinder apparatuses (details in Table 1). Six experiments at 1.0 GPa were conducted in a 3/4 inch pressure vessel at the Guangzhou Institute of Geochemistry, while one experiment at 2.5 GPa (run MORB-L2) used a 1/2 inch vessel at the Bayerisches Geoinstitut. Each assembly consisted of an outer NaCl/talc + Pyrex sleeve and a tapered graphite heater (NaCl used in Guangzhou, talc in Bayreuth). The sample capsule, housed in a pyrophyllite or MgO sleeve, was positioned at the center of the heater within Al_2_O_3_ spacers. The hot piston-in method applied pressure, automatically regulated throughout the experiment. Pressure values were further corrected for friction based on the specific assembly used (3% for NaCl + Pyrex, 18% for talc + Pyrex). Temperature control was achieved using Pt/Pt_90_Rh_10_ thermocouples and a Eurotherm controller, maintaining a deviation of ± 2 °C from the nominal temperature during the experiment. An estimated uncertainty of ± 15 °C was considered due to the temperature gradient within the capsule. Quenching was achieved by rapid cooling upon switching off the power. Each capsule was then carefully extracted from the assembly, mounted in epoxy, and polished for subsequent optical and chemical analyses.

### EPMA and LA-ICP-MS

Major element and CuO concentrations in both minerals and quenched glasses were determined using electron probe microanalysis (EPMA) on two JEOL JXA microprobes: the JXA-8100 at the Guangzhou Institute of Geochemistry and the JXA-8200 at the Bayerisches Geoinstitut. The analysis employed a focused beam for minerals and a 20 µm beam for quenched glasses. The accelerating voltage was set at 15 kV for all elements in minerals and 20 nA for Cu, while other elements in quenched glasses were analyzed at 10 nA. Counting times were set at 20 s for all elements, except Na, K, and Cu, where 10 s on the peaks for Na and K, and 40 s on the peak for Cu were used. The detection limit for Cu was approximately 350 ppm. Wavelength-dispersive spectrometry (WDS) was employed for analysis, and the PAP matrix correction was applied to raw data. Standards used included andradite (Si), MnTiO_3_ (Ti), spinel (Al), metal Fe (Fe), MnTiO_3_ (Mn), forsterite (Mg), wollastonite (Ca), albite (Na), orthoclase (K), gallium phosphite (P), metal Ni (Ni), metal Cr (Cr), and metal Cu (Cu). Excellent agreement between measurements (major elements and CuO) obtained from both instruments in Guangzhou and Bayreuth was achieved^7^.

Cu concentrations in quenched glasses were determined using laser ablation inductively coupled plasma mass spectrometry (LA-ICP-MS) at either the Bayerisches Geoinstitut (run MORB-L2) or the Guangzhou Institute of Geochemistry (run Cu-6, -45, -52, -47, and Cu-50). Both utilized 193 nm ArF excimer lasers coupled to ICP-MS systems: a Geolas M with an Elan DRC-e at Bayreuth and a Resonetic with an Agilent 7500a at Guangzhou. Laser operation parameters ranged from 5 to 10 Hz repetition rate, 80 mJ energy, and 20–80 µm spot size (typically 30 µm). The sample chamber was flushed with He at 0.4 L/min, with 5 ml/min of H_2_ added to the carrier gas to enhance Cu sensitivity. We achieved a detection limit of 0.1 ppm for Cu. NIST SRM 610 glass served as the external standard, while Si content determined by electron microprobe analysis provided the internal standard. The measured Cu concentration in the SRM 610 standard exhibited a reproducibility (1σ) of < 10%. For further details on the LA-ICP-MS methodology, please refer to Liu et al.^7^.

### XANES

The oxidation state of Cu in quenched glasses from piston cylinder experiments was investigated using Cu K-edge XANES (X-ray Absorption Near Edge Structure) spectra at the Shanghai Synchrotron Radiation Facility (BL14W1 beamline station). Measurements were conducted under well-defined beam conditions, with a stored ring electron energy of 3.5 GeV and a beam current ranging from 150 to 210 mA. The ionization chamber was filled with N_2_ to reduce attenuation and scatter. Two sets of analyses with fluorescence mode were conducted: (a) non-focusing mode: utilized a double-crystal Si (311) monochromator with a spot size of 800 × 400 µm and a 32-element high-purity Ge solid-state detector for X-ray absorption spectra collection; and (b) focusing mode: employed a Si (111) monochromator with a spot size of 200 × 200 µm and a Lytle fluorescence ionization chamber for signal collection. Both approaches effectively measured the Cu K-edge absorption edge for samples with Cu content exceeding 100 ppm.

For accurate energy calibration, the first derivative peak in the Cu foil XANES spectrum (transmission mode) was aligned to 8978.9 eV before sample measurements. The scanning range spanned 180 eV pre-edge to 200 eV post-edge, optimizing extended edge analysis for Cu coordination, particularly oxidation state. CuO_2_ and CuO standards were finely powdered and tape-mounted for transmission measurement. Spectra were recorded from 8778.9 to 9175.2 eV with a step size of 5 eV up to 8959 eV, 0.3 eV between 8959 and 9030 eV, 1 eV between 9030 and 9079 eV, and 2 eV above 9079 eV. The count time was 2 s per point. Signals from the 32-element detector were averaged, and resulting spectra were normalized using ATHENA. A total of seven samples underwent analysis through Cu K-edge XANES spectra. The first set of samples, analyzed using the non-focusing mode, comprised runs Cu-47, Cu-8, MORB-L2, Cu-6, and Cu-52. In contrast, the second set, which employed the focusing mode, included samples Cu-45, Cu-50, and Cu-52. To ensure the reliability of the results, each sample underwent multiple measurements, minimizing potential interference in the analysis process.

### Supplementary Information

Supplementary Information.

## Data Availability

The datasets used and/or analyzed during the current study available from the corresponding author on reasonable request.
