# Supplementary material for: Oxidation state of Cu in silicate melts at upper mantle conditions
Source: Sci Rep. 2024 Mar 9;14:5802. doi: 10.1038/s41598-024-56538-9 (PMC10925016; doi:10.1038/s41598-024-56538-9)
Supplement: Supplementary file 1 — Supplementary Information. [file 41598_2024_56538_MOESM1_ESM.pdf]

Supplementary materials

Table S1. Composition of the synthetic starting materials (wt%)

(wt%)	Komatiite	MORB	$Di_{70}An_{30}$
n	8	7	8
SiO_2	45.14(36)	50.07(58)	51.18(54)
TiO_2	0.45(04)	1.66(05)	0.03(02)
Al_2O_3	10.31(12)	15.38(15)	13.28(18)
FeO	11.84(27)	10.8(24)	0.06(02)
MnO	0.17(01)	0.02(01)	0.03(02)
MgO	20.35(22)	6.80(11)	10.00(11)
CaO	9.04(09)	11.61(19)	25.33(31)
Na_2O	0.77(04)	2.83(11)	0.04(03)
K_2O	0.06(02)	0.23(02)	0.04(01)
P_2O_5	0.08(01)	0.30(02)	0.02(01)
NiO	0.23(03)	0.03(01)	0.02(02)
Cr_2O_3	0.50(03)	0.01(01)	0.03(01)
Total	98.95(62)	99.74(97)	100.06(94)

Note: Data sourced from Liu et al. (2014). Compositions were determined using Electron Microprobe (EMP), with all Fe expressed as FeO.
n: number of analyses. Numbers in parentheses represent the 1σ standard deviation.

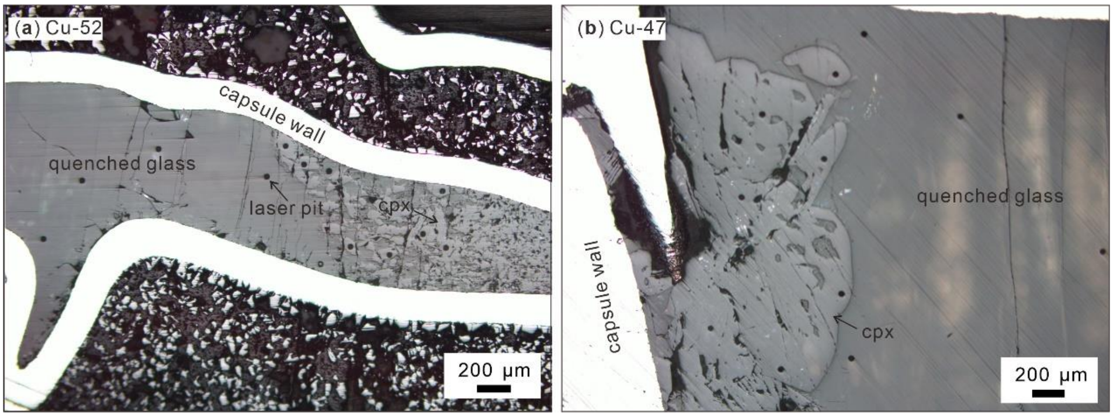

Figure S1. Reflected light images of representative run products in run Cu-52 (a) and Cu-47 (b). The diopsides (cpx) occurred at the bottom of the capsule in these runs, leaving the silicate melt pooled at the top.
